# Bub1 kinase- and H2A phosphorylation-independent regulation of Shugoshin proteins under glucose-restricted conditions

**DOI:** 10.1038/s41598-019-39479-6

**Published:** 2019-02-26

**Authors:** Yuki Kobayashi, Shigehiro A. Kawashima

**Affiliations:** Graduate School of Pharmaceutical Sciences, The University of Tokyo, 7-3-1 Hongo, Bunkyo-ku, Tokyo, 113-0033 Japan

## Abstract

Shugoshin family proteins are involved in various aspects of chromatin regulations, such as chromosome segregation, chromatin structure, and gene expression. In growing yeast and mammalian cells, C-terminal phosphorylation of histone H2A by Bub1 kinase is essential for the localization of Shugoshin proteins to chromatin. Here, we show that in stationary-phase cells, Bub1-mediated H2A phosphorylation is not necessary for chromatin localization of the Shugoshin paralog Sgo2 in *Schizosaccharomyces pombe*, or for Sgo2-dependent suppression of gene expression in subtelomeric regions. The conserved C-terminal basic domain of Sgo2, which directly binds with phosphorylated H2A, is also dispensable for the localization of Sgo2 to chromatin at stationary phase. Instead, we found that the conserved N-terminal coiled-coil domain and the uncharacterized medial region of Sgo2 are required for Bub1-independent localization of Sgo2. Moreover, Set2-mediated H3K36 methylation was important for the regulation. Intriguingly, the chromatin localization of Sgo2 in the absence of Bub1 was also observed when cells were grown in low-glucose medium. These findings suggest a novel mechanism between nutrient availability and regulation of chromatin by Shugoshin proteins.

## Introduction

Shugoshin-family proteins are widely conserved across eukaryotes and are involved in various aspects of chromatin regulation^1,2^. During mitosis and meiosis, Shugoshin proteins localize at the centromere and have at least two functions to ensure faithful chromosome segregation. First, Shugoshin complexes with protein phosphatase 2A (PP2A) to protect sister-chromatid cohesion at centromeres during the first meiotic division, or in the case of animal cells, during mitotic prophase^3,4^. Second, Shugoshin forms complexes with the chromosomal passenger complex (CPC) to ensure the bipolar attachment of kinetochores^5–7^. During interphase, one of the two Shugoshin paralogs in *Schizosaccharomyces pombe* (*S. pombe* or fission yeast), Sgo2, localizes at subtelomeres and is involved in the formation of highly condensed chromatin, suppression of expression of subtelomeric genes, and regulation of the timing of DNA replication at subtelomeric late origins^2,8–10^.

**Figure 1:**
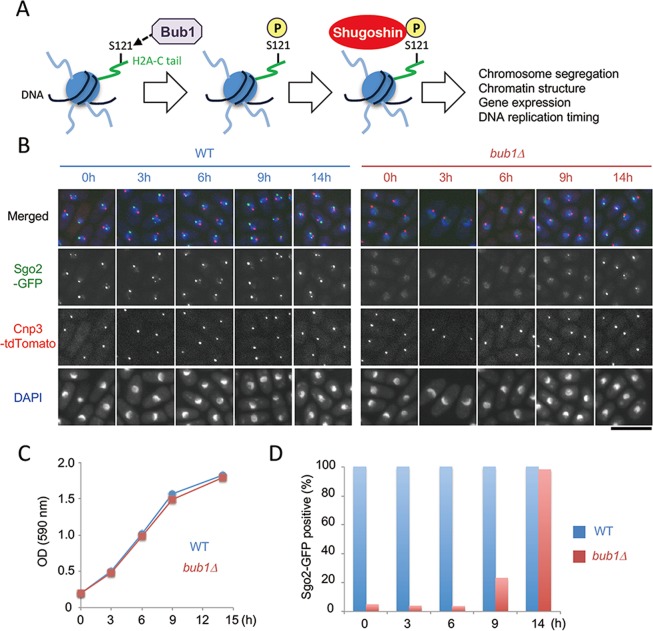
Time-course analysis of Sgo2-GFP signals in fission yeast. (**A**) Model for Bub1-dependent chromatin localization of Shugoshin in growing cells. Bub1 phosphorylates histone H2A at S121, leading to recruitment of Shugoshin to chromatin. (**B**–**D**) Time-course analysis of Sgo2-GFP in wild-type and *bub1Δ* cells. (**B**) Representative images are shown. Cnp3-tdTomato signals indicate kinetochores. DNA was stained with DAPI. Scale bar, 10 μm. (**C**) OD (590 nm) values during time-course analysis are shown. (**D**) The percentage of cells showing dot Sgo2-GFP signals were analyzed (n > 100).

In growing fission yeast, budding yeast, and human cells, the chromatin localization of Shugoshin at centromeres and subtelomeres, which is critical for its functions, depends entirely on Bub1^8,9,11–13^, a conserved serine/threonine protein kinase and reported human tumor suppressor^14^. The N-terminal non-kinase domain of Bub1 recruits spindle assembly checkpoint components to the kinetochores^15^, whereas the C- terminal kinase domain of Bub1 catalyzes histone H2A (serine 121) phosphorylation in *S. pombe* and *S. cerevisiae* (or T120 in human) and recruits Shugoshin onto chromatin (Fig. 1A)^9,13,16,17^. Other proteins that regulate Shugoshin localization include Bir1 and Set2. Bir1 is a subunit of CPC and is required for the centromeric localization of Sgo2 during mitosis or meiosis in fission yeast^5^. Set2 is a methyltransferase for histone H3-K36 and is required for the subtelomeric localization of Sgo2 during interphase in growing fission yeast cells^13^. Shugoshin family proteins share two conserved domains^8^. The N-terminal coiled-coil domain is required for dimerization or interaction with PP2A^18^ or Bir1^7^. The C-terminal basic domain directly binds with H2A-phosphorylated nucleosomes^17^, and is thus essential for chromatin localization of Shugoshin in growing cells^9,16^. The medial region of Shugoshin is not well conserved across eukaryotes, therefore its functions may have diverged among Shugoshin family proteins.

Here, we show that Bub1-mediated H2A phosphorylation is not necessary for the localization of Sgo2 to chromatin or for the function of Sgo2 in gene expression in stationary-phase fission yeast cells. Glucose restriction also allows Sgo2 localization to chromatin in the absence of Bub1. Our findings indicate a previously unknown link between nutrient availability and regulation of chromatin by Shugoshin proteins.

## Results

### Bub1-mediated H2A phosphorylation is dispensable for the localization of Sgo2 to chromatin in stationary-phase fission yeast cells

In growing fission yeast cells, Sgo2 mainly associates with subtelomeres of chromosomes 1 and 2 during interphase and with centromeres during mitosis^5,9,13^. Therefore, Sgo2-green fluorescent protein (GFP) signals appeared as one to several dots under a fluorescence microscope (Fig. 1B, wild type [WT]). Because H2A phosphorylation by Bub1 is essential for Sgo2 localization^9,13^, Sgo2-GFP signals were rarely observed in log-phase *bub1Δ* cells (Fig. 1B, *bub1Δ*, 0–6 h). However, during time-course analysis of Sgo2-GFP signals in *bub1Δ* cells, we found that Sgo2-GFP dots were observed as cultures approach stationary phase (Fig. 1B–D). While <5% *bub1Δ* cells show Sgo2-GFP signals from 0 (A_590nm_ = 0.2) to 6 h (A_590nm_ ≈ 1.0), nearly all *bub1Δ* cells showed Sgo2-GFP signals at 14 h (A_590nm_ ≈ 1.8) (Fig. 1B–D).

**Figure 2:**
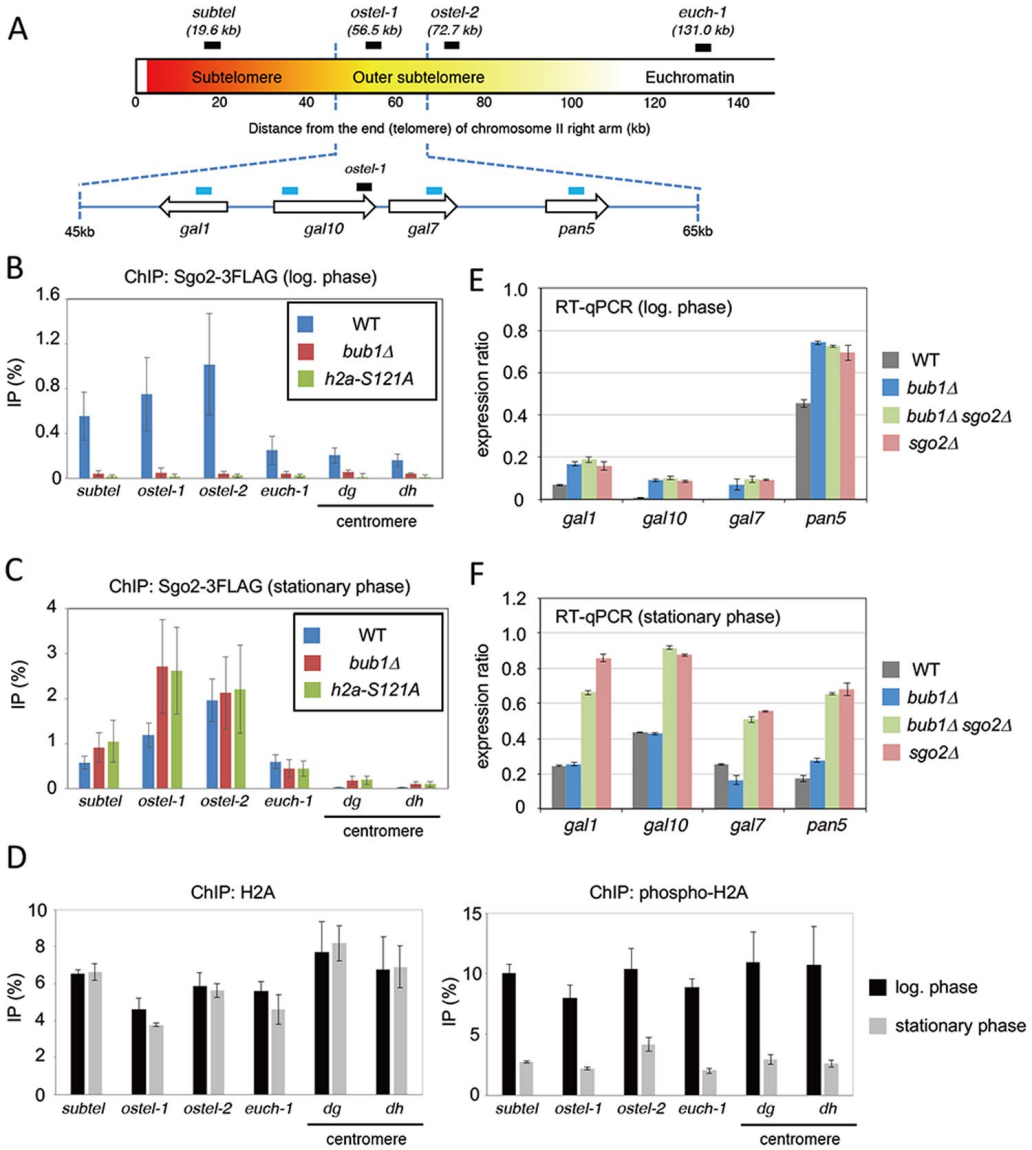
Bub1-mediated H2A phosphorylation is dispensable for chromatin localization and function of Sgo2 in stationary-phase cells. (**A**) Schematic illustration of *S. pombe* chromosome II right arm. The location of primer sets for ChIP experiments (black) or reverse transcription quantitative PCR (RT-qPCR) experiments (blue) are shown. (**B**–**C**) ChIP analyses of Sgo2-3FLAG localization in WT, *bub1Δ*, and *h2a-S121A* cells at the log phase (**B**) or stationary phase (**C**). The graph shows the percentage of co-immunoprecipitated DNA with anti-FLAG antibody per total DNA in whole cell extracts (error bar = SD of more than 3 experiments). (**D**) ChIP analyses of H2A or H2AS121ph (phospho-H2A) localization in wild-type cells at the log phase or stationary phase. The graph shows the percentage of co-immunoprecipitated DNA with anti-H2A or anti-H2AS121ph antibody per total DNA in whole cell extracts (error bar = range of 2 experiments). (**E**–**F**) The expression of *gal1*^+^, *gal10*^+^, *gal7*^+^, and *pan5*^+^ genes in the indicated strains at the log phase (**E**) or stationary phase (**F**) was analyzed by RT-qPCR (error bar = range of 2 experiments).

The Sgo2-GFP dot signals did not co-localize with those of Cnp3-tdTomato, which localized at kinetochores (Fig. 1B). To determine chromosomal regions in which Sgo2 is enriched in *bub1Δ* cells at stationary phase, we performed chromatin immunoprecipitation (ChIP) analyses using *sgo2-3FLAG* strains. We chose four representative regions (*subtel*, *ostel-1*, *ostel-2*, and *euch-1*) within a ~140 kb region at the end of the right arm of chromosome II (Fig. 2A). The *subtel* region (“Subtelomere” in Fig. 2A) resides in a heterochromatin region containing histone H3-K9 methylation and the heterochromatin-associated protein Swi6^19,20^. The *ostel-1* and *ostel-2* regions (“Outer subtelomere” in Fig. 2A) reside in a heterochromatin-adjacent region in which the Sgo2-dependent highly condensed chromatin bodies or knobs form^10,13^. The *euch-1* locus resides outside the outer subtelomeric region. In addition, enrichment at centromeric regions (*dg*, *dh*) was examined. Consistent with previous reports^9,13^, Sgo2 associated with the subtelomere mainly at the *ostel-1* and *ostel-2* regions in a Bub1-dependent manner in log-phase cells (Fig. 2B). We found that in stationary phase cells, however, Sgo2 association at subtelomeres was independent of Bub1 (Fig. 2C). Substituting serine 121 of H2A with a non-phosphorylable alanine (*h2a-S121A*) resulted in almost the same chromatin association of Sgo2 as observed in *bub1Δ* cells (Fig. 2B,C). The level of Sgo2-3FLAG was comparable between log-phase and stationary-phase in wild-type, *bub1Δ*, or *h2a-S121A* cells (Fig. S1A). These data indicate that Bub1-mediated H2A phosphorylation is not necessary for the localization of Sgo2 to chromatin in stationary-phase fission yeast cells. We also performed ChIP analysis to compare the relative amounts of H2A phosphorylation at serine 121 between the log phase and stationary phase in wild-type cells. While H2A was comparably abundant between the two phases, the abundance of phosphorylated H2A was significantly lower in stationary-phase cells compared with log-phase cells (Fig. 2D). This result further supports that Sgo2 chromatin localization at stationary phase is independent of H2A phosphorylation.

### Sgo2, but not Bub1, suppresses gene expression in subtelomeric regions in stationary-phase cells

Sgo2 represses transcription of the subtelomeric genes in a Bub1- and H2A phosphorylation-dependent manner in growing fission yeast cells^13^. To examine whether the transcription of subtelomeric genes in stationary-phase cells is also regulated by Sgo2 or Bub1, we chose four representative genes (*gal1*, *gal10*, *gal7*, and *pan5*) that are located at 45–65 kb from the end of the right arm of chromosome II (Fig. 2A) and examined their expression in log-phase and stationary-phase cells. Consistent with a previous report^13^, all four genes were more highly expressed in both *sgo2Δ* cells and *bub1Δ* cells at the log phase (Fig. 2E). Double deletion of Sgo2 and Bub1 did not further increase the expression of these genes (Fig. 2E), indicating that Bub1 represses transcription of these subtelomeric genes through Sgo2. Transcripts of *gal1*, *gal10*, and *gal7* were relatively more abundant in stationary-phase cells than in log-phase cells, whereas those of *pan5* were relatively less abundant (Fig. 2F). This result suggests that *gal*1/7/10 and *pan5* expression was differentially regulated in response to nutrient restriction. However, *sgo2* deletion increased the expression of all four genes in stationary-phase cells (Fig. 2F). On the other hand, *bub1* deletion did not affect the expression of any of these four genes in stationary-phase cells (Fig. 2F). These data suggest that Bub1 is not necessary for the Sgo2-dependent suppression of expression of genes in the subtelomeric regions in stationary-phase cells, and that Sgo2-dependent transcriptional regulation is independent of transcriptional changes that depend on nutrient restriction.

### Set2-mediated H3K36 methylation is essential for Bub1-independent association of Sgo2 with subtelomeres in stationary-phase cells

**Figure 3:**
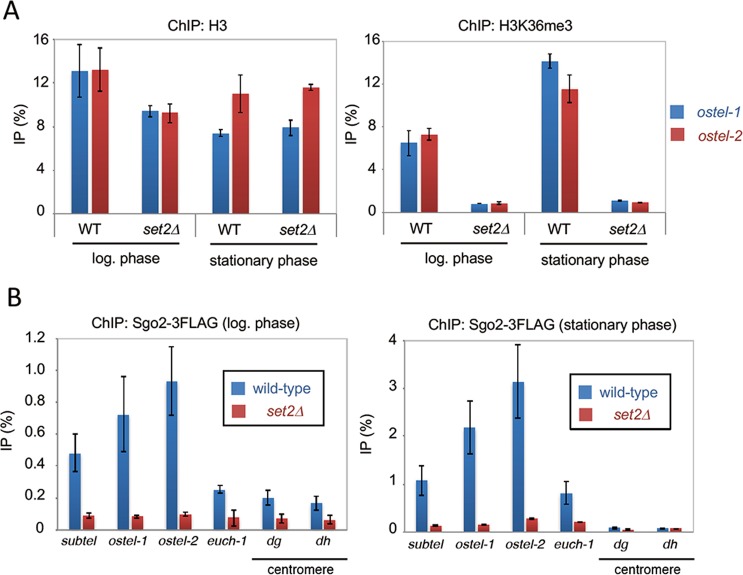
Set2 is required for Bub1-independent localization of Sgo2 to chromatin. (**A**) ChIP analyses of H3 or H3K36me3 localization in wild-type or *set2Δ* cells at the log phase or stationary phase. The graph shows the percentage of co-immunoprecipitated DNA with anti-H3 or anti- H3K36me3 antibody per total DNA in whole cell extracts (error bar = range of 2 experiments). (**B**) ChIP analyses of Sgo2-3FLAG localization in WT or *set2Δ* cells at the log phase (left) or stationary phase (right). The graph shows the percentage of co-immunoprecipitated DNA with anti-FLAG antibody per total DNA in whole cell extracts (error bar = range of 2 experiments).

We then explored which factors are important for Bub1-independent association of Sgo2 with subtelomeres in stationary-phase cells. Set2 is the methyltransferase for H3-K36 in fission yeast^21,22^. It has been shown that Set2-mediated H3-K36 methylation is essential for the formation of highly condensed chromatin and is also required for Sgo2-dependent transcription of gene located in subtelomeric regions^10,13^. Consistent with these earlier studies, our ChIP data using log-phase cells showed that Sgo2 association and the degree of methylation of H3-K36 at subtelomeres depends on Set2 (Fig. 3A,B). We then examined the degree of H3-K36me3 in stationary-phase cells. Our ChIP analyses of histone H3 and H3-K36me3 showed that the level of H3K36me3 at *ostel-1* and *ostel-2* depends on Set2 in stationary-phase cells (Fig. 3A). Intriguingly, we found that subtelomeric localization of Sgo2 in stationary-phase cells was also drastically decreased in *set2Δ* cells (Fig. 3B). The level of Sgo2-3FLAG was comparable between log-phase and stationary-phase in wild-type or *set2Δ* cells (Fig. S1A). These data suggest that Set2-mediated H3-K36 methylation is required for Bub1-independent association of Sgo2 with subtelomeres in stationary-phase cells.

### The conserved N-terminal coiled-coil domain, but not the conserved C-terminal basic domain, of Sgo2 is involved in Bub1-independent localization of Sgo2 to chromatin

**Figure 4:**
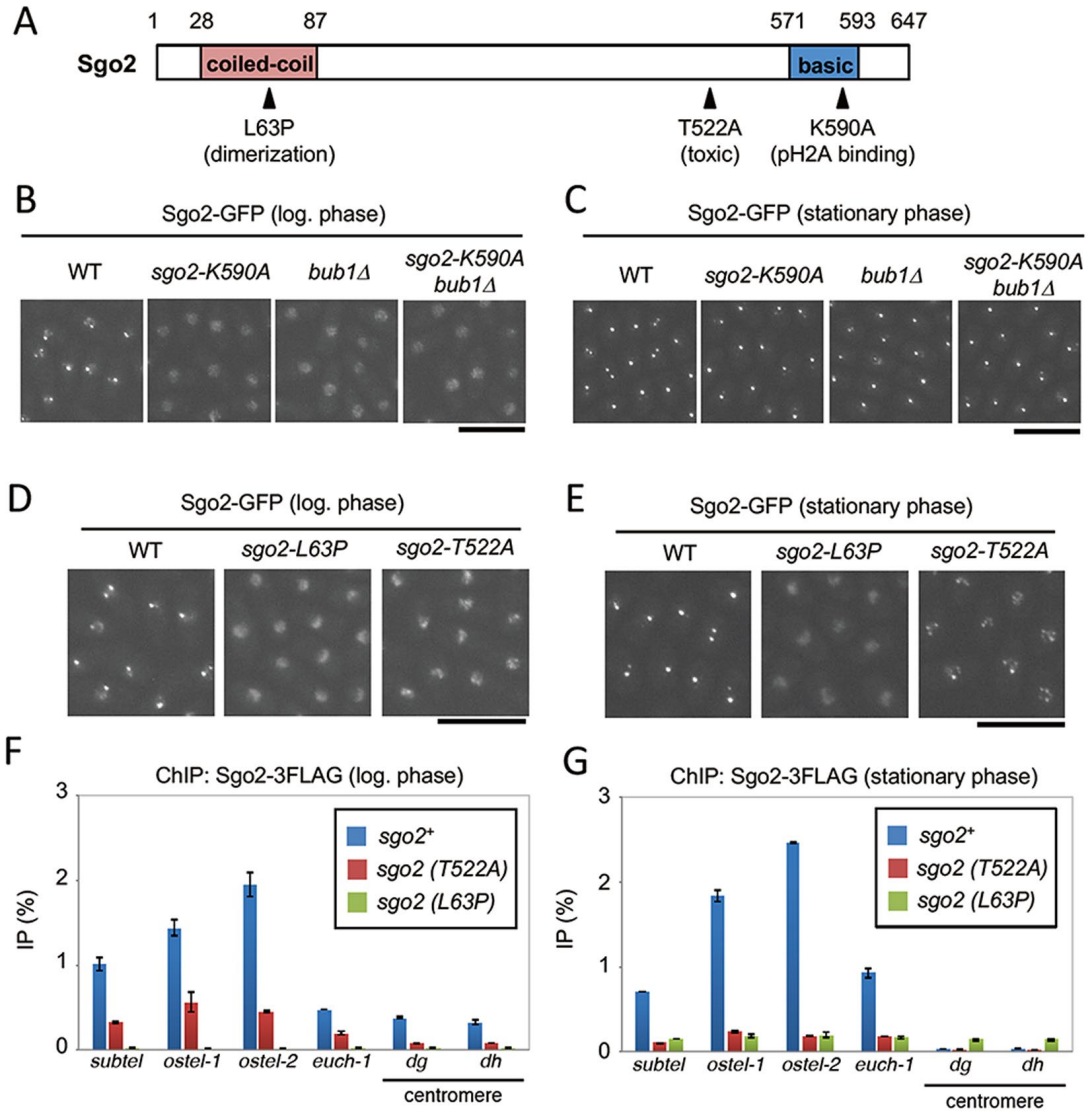
Analyses of localization of Sgo2 mutants. (**A**) Schematic illustration of Sgo2. (**B**,**C**) Sgo2-GFP signals in WT, *sgo2-K590A*, *bub1Δ*, and *sgo2-K590A bub1Δ* cells at the log phase (**B**) or stationary phase (**C**) were examined. Scale bar, 10 μm. (**D**,**E**) Sgo2-GFP signals in WT, *sgo2-L63P*, and *sgo2-T522A* cells at the log phase (**D**) or stationary phase (**E**) were examined. Scale bar, 10 μm. (**F,G**) ChIP analyses of Sgo2-3FLAG localization in wild-type (*sgo2*^+^), *sgo2-L63P*, or *sgo2-T522A* cells at the log phase (**F**) or stationary phase (**G**). The graph shows the percentage of co-immunoprecipitated DNA with anti-FLAG antibody per total DNA in whole cell extracts (error bar = range of 2 experiments).

The N-terminal coiled-coil domain and the C-terminal basic domain of Sgo2 are highly conserved among Shugoshin family proteins (Fig. 4A)^8^. The basic domain directly interacts with phosphorylated H2A (pH2A) in nucleosomes^17^, and is thus essential for chromatin localization of Shugoshin in growing cells^9,16^. Substituting lysine 590 of Sgo2 with alanine (*sgo2-K590A*), which reduces the cationic property of the basic domain, resulted in abnormal diffuse localization of Sgo2 in log-phase *bub1Δ* cells (Fig. 4B). In contrast, Sgo2 (K590A)-GFP showed dot-like signals in stationary-phase cells, as did Sgo2 (WT)-GFP (Fig. 4C), indicating that the basic region of Sgo2 is not required for its Bub1-independent chromatin localization in stationary-phase cells. The coiled-coil domain of Shugoshin proteins is required for their dimerization or interaction with PP2A or Bir1^7,18^. Two-hybrid analysis showed that the Sgo2N2 fragment (aa1–206) that contains the coiled-coil domain could form dimers, and that substitution of leucine 63 of Sgo2 with proline (L63P) abolished this dimerization (Fig. 4A, Fig. S2A). Strikingly, Sgo2 (L63P)-GFP showed abnormal diffuse localization in both log-phase and stationary-phase cells (Fig. 4D,E). ChIP analysis using *sgo2-3FLAG* strains also showed that subtelomeric localization of Sgo2 was significantly reduced in the *sgo2-L63P* mutant even at stationary phase (Fig. 4F,G). The level of Sgo2-3FLAG was comparable in wild-type (*sgo2*^+^), *sgo2-T522A*, or *sgo2-L63P* cells (Fig. S1B). These data indicate that the coiled-coil domain of Sgo2 is indispensable for its Bub1-independent chromatin localization. The medial region of Shugoshin is not well conserved across eukaryotes; therefore, its functions have likely diverged among Shugoshin family proteins. We found that overexpression of a Sgo2-T522A mutant significantly inhibited cell growth, compared with Sgo2 (WT) (Figs 4A, S2B). This inhibitory effect was likely independent of Bub1 and H2A phosphorylation, as it was not suppressed by the K590A mutation of Sgo2 (Fig. S2B). On the other hand, the L63P mutation of Sgo2 suppressed the inhibitory effect of Sgo2-T522A overexpression (Fig. S2B). Remarkably, fluorescence microscopy and ChIP analyses showed that the proportion of localization of Sgo2 (T522A) to chromatin was notably lower than that of Sgo2 (WT) at both the log and stationary phase (Fig. 4F,G), suggesting that the medial region of Sgo2 that contains T522 is also involved in its Bub1-independent chromatin localization.

### Glucose restriction suppresses the requirement for Bub1 in localization of Sgo2 to chromatin

**Figure 5:**
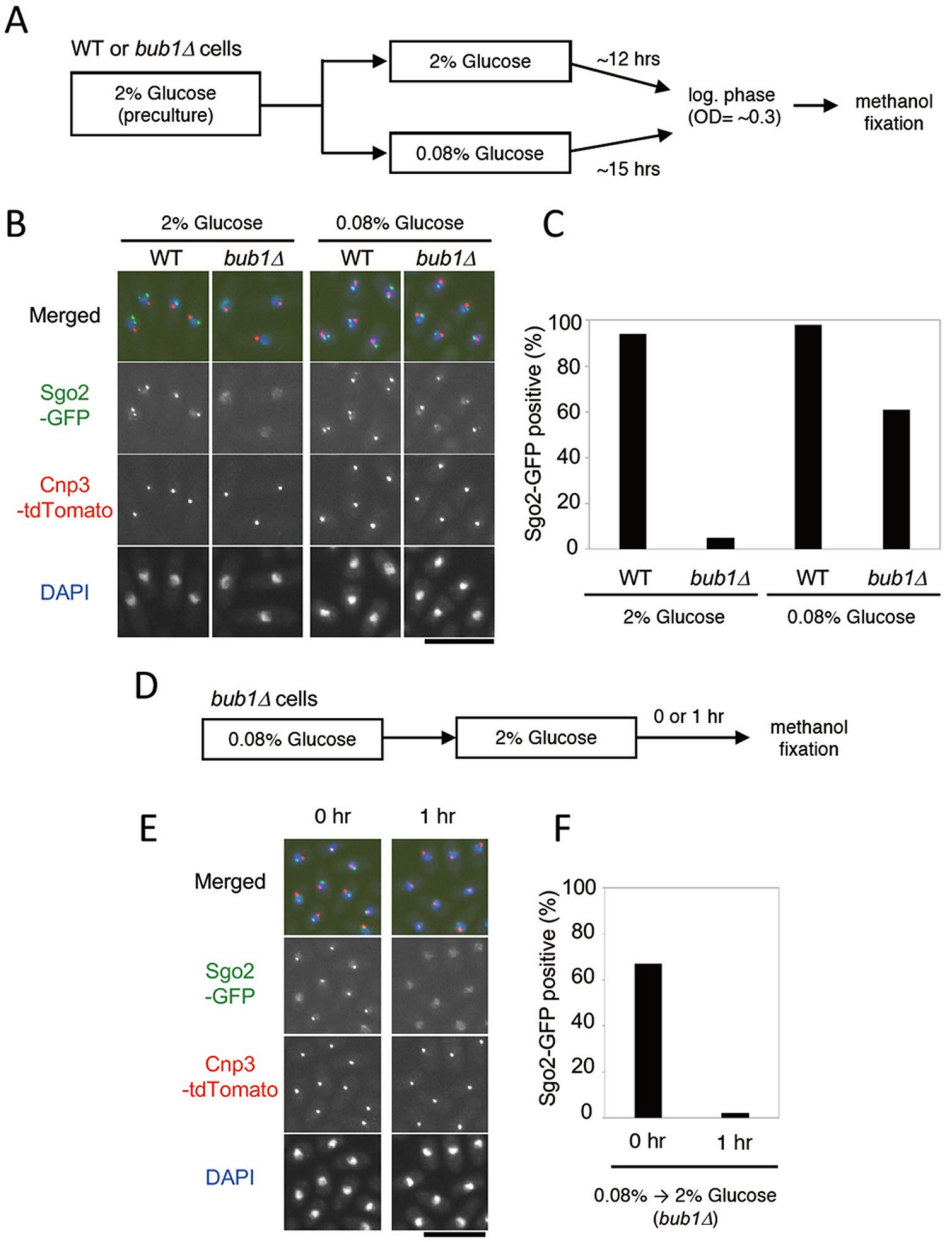
Glucose restriction suppresses the requirement of Bub1 for Sgo2 localization. (**A**–**C**) Sgo2-GFP signals in WT or *bub1Δ* cells at the log phase in medium containing 2% or 0.08% glucose were examined. (**A**) Scheme of the experiment. (**B**) Representative images are shown. Cnp3-tdTomato signals indicate kinetochores. DNA was stained with DAPI. Scale bar, 10 μm. (**C**) The percentage of cells showing dot Sgo2-GFP signals were analyzed (n > 200). (**D**–**F**) Sgo2-GFP signals in *bub1Δ* cells were examined. (**D**) Scheme of the experiment. (**E**) Representative images are shown. Cnp3-tdTomato signals indicate kinetochores. DNA was stained with DAPI. Scale bar, 10 μm. (**F**) The percentage of cells showing Sgo2-GFP signals were analyzed (n > 200).

As cultures approach stationary phase, nutrients such as glucose become gradually depleted in cells. Therefore, we hypothesized that low glucose might be a critical factor regulating Bub1-independent Sgo2 chromatin localization. The experiments described above were performed using yeast extract (YE) medium containing 2% (111 mM) glucose. Previous reports showed that *S. pombe* cells can proliferate in Edinburgh Minimal Medium (EMM) containing 0.08% (4.4 mM) glucose. When cells were transferred from medium containing 2% glucose to 0.08% glucose, the cell cycle arrested at G2 for a period of 1–2 generations, and then cell division resumed^23–27^. To address whether glucose concentration affects localization of Sgo2 to chromatin, WT and *bub1Δ* cells were cultured in EMM containing 2% or 0.08% glucose for more than 12 hour at 29 °C, and then log-phase cells (A_590nm_ ≈ 0.3) were fixed for analysis by fluorescence microscopy (Fig. 5A). As observed in YE medium, Sgo2 signals were not observed in *bub1Δ* cells in EMM with 2% glucose (Fig. 5B,C). Intriguingly, in EMM with 0.08% glucose, about 60% of *bub1Δ* cells showed Sgo2 dot-like signals (Fig. 5B,C), while the level of Sgo2-3FLAG was comparable between 2% and 0.08% glucose (Fig. S1C). Furthermore, when *bub1Δ* cells were transferred from 0.08% to 2% glucose medium and incubated for 1 h, Sgo2 dot-like signals again disappeared (Fig. 5E,F). We also examined localization of Sgo2 mutants, and found that Sgo2 (K590A)-GFP, but not Sgo2 (L63P)-GFP, showed signals in 0.08% glucose medium (Fig. S2). These data suggested that glucose restriction suppressed the requirement for Bub1 and H2A phosphorylation in localization of Sgo2 to chromatin.

## Discussion

Taken together, these data suggest that localization of Sgo2 to chromatin is regulated by glucose concentration in fission yeast cells. In high-glucose media, such as YE medium or EMM containing 2% glucose, localization of Sgo2 to chromatin depends completely on Bub1-mediatted H2A phosphorylation, as described previously^9,13^. The molecular mechanisms responsible for Bub1-dependent localization of Sgo2 to chromatin could include direct binding of phosphorylated H2A in chromatin to the basic region of Sgo2^17^. In low-glucose media, such as EMM containing 0.8% glucose, Bub1-mediated H2A phosphorylation is no longer necessary for localization of Sgo2 to chromatin. Consistent with this model, the basic region of Sgo2 was also not required. In contrast, the conserved N-terminal coiled-coil domain and the uncharacterized medial region of Sgo2 are indispensable for localization of Sgo2 to chromatin. How Sgo2 localizes to chromatin under glucose-restricted conditions is the important subsequent question. Identifying chromatin proteins that interact with Sgo2 in glucose-restricted condition may help elucidate the molecular mechanism of Bub1-independent localization of Sgo2 to chromatin.

Decreases in glucose concentration in media strongly affects gene expression in *S. pombe*^28,29^. In addition, a quantitative metabolomic approach for *S. pombe* cell extracts has revealed that the quantities of specific metabolites increase or decrease under different glucose concentrations^23^. Interestingly, some of these metabolites are cofactors of chromatin-modifying enzymes. The amount of S-adenosyl methionine (SAM), a methyl donor for the histone methyltransferases, increased dramatically under low glucose concentrations, suggesting that histone methylation is promoted by glucose restriction. Limiting glucose availability diminishes glycolysis-related metabolites^23^, leading to reduction of acetyl-CoA, an acetyl donor for the histone acetyltransferases, and histone acetylation. Therefore, the patterns of post-translational modifications (PTMs) of histones appear to change under glucose-restricted conditions. Based on the crystal structure of the nucleosome^30^, the N-terminus of histone H3 is proximal to the C-terminus of histone H2A, where the basic region of Sgo2 directly interacts. Therefore, whether Sgo2 might read histone PTMs that are specific to low glucose conditions in histone H3 N-termini *via* its conserved N-terminal coiled-coil domain and/or its uncharacterized medial region is an attractive hypothesis to be addressed in future experiments.

## Methods

### *S. pombe* strain construction and media

All strains used are listed in Table S1. PCR-based gene targeting was used to construct gene deletions and fluorescent protein-tagged strains with selectable marker gene cassettes^31^. The *sgo2* mutant plasmids were generated using a PrimeSTAR^®^ Mutagenesis Basal Kit (Takara, R046A). To generate *sgo2* mutant strains, genomic *sgo2-GFP* ≪ *hygr* or *sgo2-3FLAG* ≪ *kanr* fragments carrying the mutations were transformed into wild-type cells. Then, *sgo2-GFP* ≪ *hygr* or *sgo2-3FLAG* ≪ *kanr* mutant strains carrying selectable marker gene cassettes were selected on appropriate media and confirmed by PCR and sequencing. The *h2a-S121A* mutant strain was generated as described previously^9^. Standard yeast growth conditions and methods were used^32^. Most experiments were performed in YE medium containing adenine, leucine, uridine, and histidine. Experiments in Fig. 5 and Fig. S2 used EMM containing 2% or 0.08% glucose. Log-phase cells were harvested when the OD_590nm_ value ranged from 0.25 to 0.6. To prepare stationary-phase cells, cells (OD_590nm_ = 0.1) were cultured at 29 °C for 17 hrs.

### Image acquisition

For all microscopy experiments, cells were fixed using methanol and stained with DAPI, and images were acquired with a microscope (Axio Imager 2; Carl Zeiss) with a 63x objective (Plan-APOCHROMAT, 63×/1.4 Oil DIC; Carl Zeiss) and processed with Axio Vision 4.8 software (Carl Zeiss). A Z-stack of ~2 μm thickness, with single planes spaced by 0.3 μm, was acquired and maximum-intensity projections were generated. To compare signal intensities, all images were taken under the same exposure conditions and processing methods.

### Immunoblotting

Cells were harvested and washed using STOP buffer (150 mM NaCl, 50 mM NaF, 10 mM EDTA, 1 mM NaN_3_ (pH 8.0)). Cell pellet was mixed with equal amount of buffer 1 (50 mM Tris-HCl (pH 7.5), 1.5 mM MgCl_2_, 300 mM NaCl, 0.3% NP-40, 1 mM DTT, cOmplete^™^ Protease Inhibitor Cocktail (Sigma), PhosSTOP^™^ (Sigma)), and the mixture was beaten with glass beads using Fastprep bead-beater. Buffer 2 (50 mM Tris-HCl (pH 7.5), 1.5 mM MgCl_2_, 7.5% glycerol, 1mM DTT, cOmplete^™^ Protease Inhibitor Cocktail, PhosSTOP^™^) was added in volumes 2x the cell pellet, and the mixture was centrifuged (5k rpm, 2 min once, and 14k rpm, 10 min twice) to obtain supernatants (whole cell extracts). Whole cell extracts were separated by SDS-PAGE using 5–20% gels, and transferred to PVDF membranes (Immobilon P, Millipore). 5% skim milk in TBST was used for blocking. The following antibodies were used. Primary antibodies: anti-FLAG (Sigma, F1804, 1:1,000); anti-tubulin (TAT1^33^). Secondary antibodies: anti-mouse IgG-HRP (GE, NA931V, 1:10,000). Chemiluminescence was generated by Luminata Forte HRP substrate (Millipore) and detected by LAS 4000 mini (GE).

### ChIP (Chromatin immunoprecipitation)

Log- or stationary-phase cells at 29 °C were fixed in formaldehyde solution (11% formaldehyde, 50 mM Tris-HCl (pH 7.5), 1 mM EDTA (pH 8.0), 0.5 mM EGTA, 100 mM NaCl) for 10 min at 29 °C and for more than 50 min at 4 °C. After washing four times with Buffer 1 (50 mM HEPES (pH 7.5), 1 mM EDTA, 140 mM, NaCl, 1% Triton X-100, 0.1% Na deoxycholate), pellets were frozen in liquid nitrogen and stored at −80 °C. Cell pellet was mixed with equal amount of buffer 1, and the mixture was beaten with glass beads using Fastprep bead-beater. After cells were lysed by sonication in an Ultrasonic Disruptor (TOMY Digital Biology), the supernatants (WCE: whole cell extract) were collected. For Sgo2-3FLAG IPs, after 300 µL of WCE (1.2 mg/mL) was incubated with anti-FLAG or normal mouse IgG (Santa Cruz, sc-2025) for 30 min on ice, Dynabeads^®^ Protein G (20 µL, Veritas) was added to the WCE-antibody mixture and incubated for 2 hrs at 4 °C. For H2A, phosphor-H2A, histone H3, and H3-K36me3 IPs, after 150 µL of WCE (1.2 mg/mL) was incubated with anti-H2A (active motif, 39945), anti-H2AS121ph^9^, anti-H3 (abcam, ab1791), anti-H3K36me3 (abcam, ab9050), or normal rabbit IgG (Santa Cruz, sc-2027) antibodies for 30 min on ice, 10 µL of Dynabeads Protein G were added to the WCE-antibody mixture and incubated for 2 hrs at 4 °C. After washing in Buffer 1, Buffer 1’ (50 mM HEPES (pH 7.5), 500 mM, NaCl, 1 mM EDTA, 1% Triton X-100, 0.1% Na deoxycholate), Buffer 2 (40 mM Tris-HCl (pH 8.0), 250 mM LiCl, 0.5% NP-40, 0.5% Na deoxycholate), and TE, the co-immunoprecipitated DNA was extracted for 15 min at 65 °C with Elution buffer (20 mM Tris-HCl (pH 7.5), 100 mM NaCl, 20 mM EDTA, 0.1% SDS). The supernatant was collected, incubated at 75 °C overnight, and incubated with Proteinase K (Takara, 9034) for 3 hrs at 55 °C. Then, DNA was purified by PCR Clean-Up Mini Kit (Favorgen) before performing PCR. DNA prepared from WCE or immunoprecipitated fractions was analyzed by quantitative PCR using LightCycler^®^ 480 system (Roche) with SYBR^™^ Green I Master (Roche). The amount of co-immunoprecipitated DNA was divided by the amount of total DNA in WCE to calculate IP (%) of each antibody. Then, the final IP (%) for each antibody (%), shown on the *y*-axis of the graph, was calculated by subtracting IgG IP (%) from the total IP (%). Primers used in ChIP assays are listed in Table S2.

### Reverse transcription quantitative PCR analysis

Total RNA was isolated from fission yeast using a hot phenol method followed by phenol-chloroform extraction and precipitation^34^. After remaining DNA was digested with DNase I (Takara, 2270A) in buffer containing 40 mM Tris-HCl, 8 mM MgCl_2_, 1 mM CaCl_2_, and 5 mM DTT, the RNA was further purified on an RNeasy^®^ Mini spin column (Qiagen) following the manufacturer’s instructions. Reverse transcription was carried out using 1 µg total RNA template, following the manufacturer’s protocol in the presence or absence of enzyme (SuperScript^™^ III First-Strand Synthesis System, Invitrogen). For quantitative PCR, SYBR Green (Applied Biosystems) and primers were mixed, and the starting quantity of DNA was estimated from the number of cycles (Ct value) required to reach the threshold using a Roche LightCycler 480 System. Primers used in this study are listed in Table S3.

## Supplementary information

Supplementary information
