# Supplementary material for: Bub1 kinase- and H2A phosphorylation-independent regulation of Shugoshin proteins under glucose-restricted conditions
Source: Sci Rep. 2019 Feb 26;9:2826. doi: 10.1038/s41598-019-39479-6 (PMC6391426; doi:10.1038/s41598-019-39479-6)
Supplement: Supplementary file 1 — Supplementary information [file 41598_2019_39479_MOESM1_ESM.pdf]

Supplementary Information for:

Bub1 kinase- and H2A phosphorylation-independent regulation of Shugoshin proteins under glucose-restricted conditions

Yuki Kobayashi and Shigehiro A. Kawashima*

Graduate School of Pharmaceutical Sciences, The University of Tokyo, 7-3-1 Hongo, Bunkyo-ku, Tokyo 113-0033, Japan

Corresponding Author:
Shigehiro A. Kawashima
Email: skawashima@mol.f.u-tokyo.ac.jp

Contents:

1. Supplementary Figures
2. Supplementary Tables

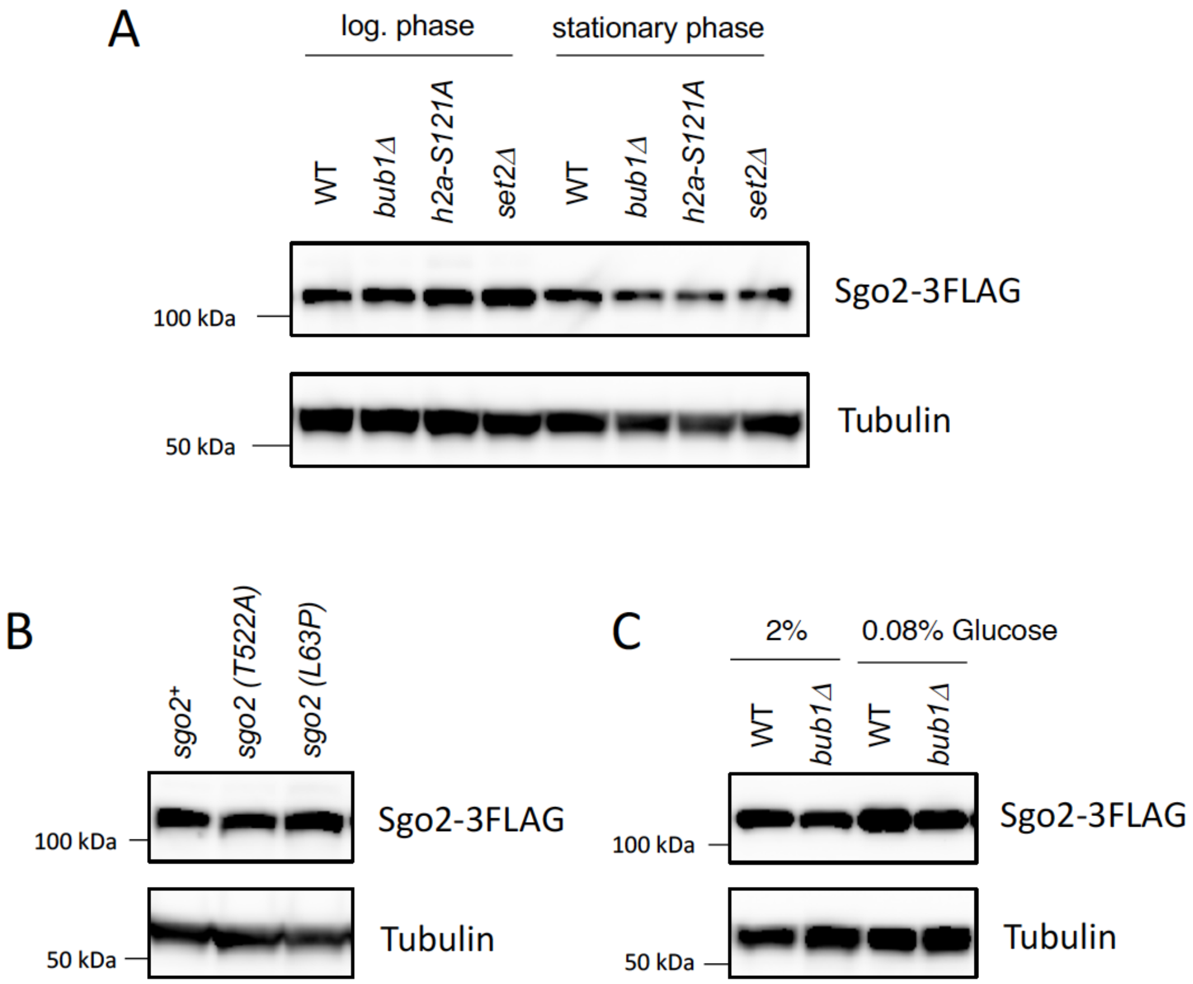

Figure S1. The level of Sgo2-3FLAG in the indicated strains was estimated by western blotting with anti-FLAG antibody. Tubulin is the loading control. (A) log-phase vs stationary phase in YE medium. (B) log-phase in YE medium. (C) EMM medium with 2% or 0.08 % glucose.

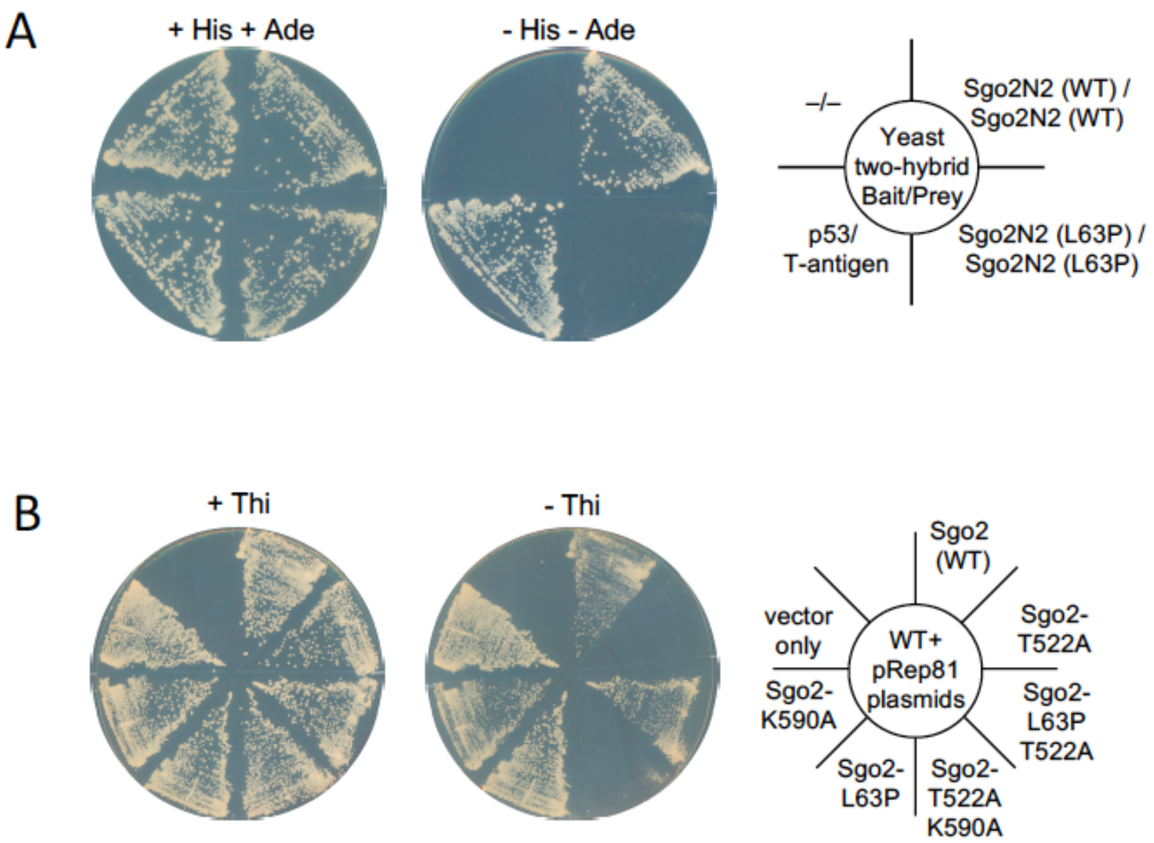

Figure S2. Additional analysis of Sgo2 mutants. (A) Yeast two-hybrid assay for dimerization of the Sgo2N2 peptide (aa1–206). The p53/T-antigen was used as a positive control. (B) Cells in which wild-type Sgo2 (WT), the indicated Sgo2 mutant, or vector control (pRep81) were overexpressed from the nmt81 promoter were streaked on plates. Thiamine (Thi) addition represses transcription from the nmt81 promoter.

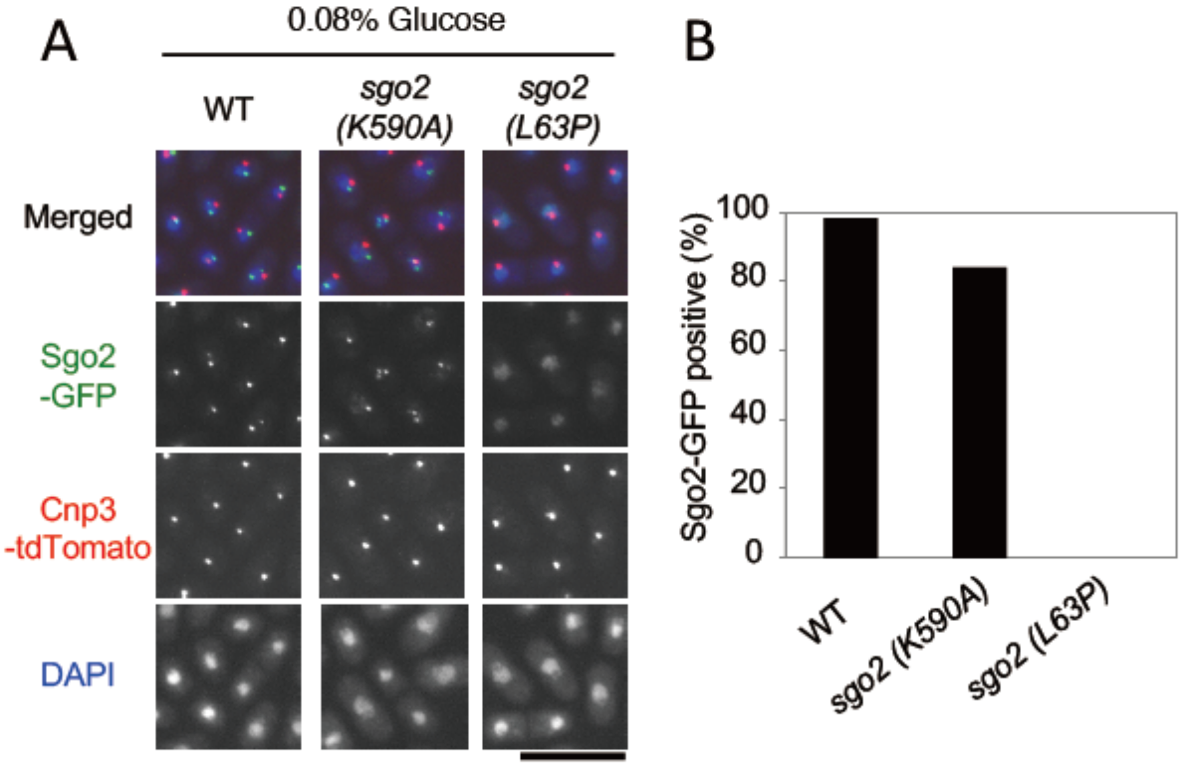

Figure S3. Analysis of Sgo2 mutants under glucose-restricted conditions. (A–B) Sgo2-GFP signals in WT, *sgo2-L63P*, or *sgo2-T522A* cells at the log phase in medium containing 0.08% glucose were examined. (A) Representative images are shown. Cnp3-tdTomato signals indicate kinetochores. DNA was stained with DAPI. Scale bar, 10 μm. (B) The percentage of cells showing Sgo2-GFP signals were analyzed (n >200).

Table S1. Strain list

Fig. 1	SAK856	h-	cnp3-tdtomato<<kanr sgo2-GFP<<hygr
	SAK885	h-	cnp3-tdtomato<<kanr sgo2-GFP<<hygr bub1::natr
Fig. 2B, C	SAK3672	h-	sgo2-3flag<<kanr
	SAK3745	h-	ade6 leu1 bub1::ura4+ sgo2-3flag<<kanr
	SAK3746	h-	ade6 leu1 hta1(S121A) hta2(S121A) sgo2-3flag<<kanr
Fig. 2D	SAK3672	h-	sgo2-3flag<<kanr
Fig. 2E, F	SAK3755	h90	
	SAK3758	h90	bub1::natr
	SAK3768	h90	sgo2::bsdr bub1::natr
	SAK3769	h90	sgo2::bsdr
Fig. 3	SAK3672	h-	sgo2-3flag<<kanr
	SAK3824	h-	sgo2-3flag<<kanr set2::natr
Fig. 4B, C	SAK856	h-	cnp3-tdtomato<<kanr sgo2-GFP<<hygr
	SAK885	h-	cnp3-tdtomato<<kanr sgo2-GFP<<hygr bub1::natr
	SAK3843	h90	cnp3-tdtomato<<kanr sgo2(590KA)-GFP<<hygr
	SAK3847	h+ or h-	cnp3-tdtomato<<kanr bub1::natr sgo2(590KA)-GFP<<hygr
Fig. 4D, E	SAK856	h-	cnp3-tdtomato<<kanr sgo2-GFP<<hygr
	SAK3840	h90	cnp3-tdtomato<<kanr sgo2(522TA)-GFP<<hygr
	SAK3848	h90	cnp3-tdtomato<<kanr sgo2(63LP)-GFP<<hygr
Fig. 4F, G	SAK3672	h-	sgo2-3flag<<kanr
	SAK3794	h+	sgo2(522TA)-3flag<<kanr
	SAK3797	h+	sgo2(63LP)-3flag<<kanr
Fig. 5	SAK856	h-	cnp3-tdtomato<<kanr sgo2-GFP<<hygr
	SAK885	h-	cnp3-tdtomato<<kanr sgo2-GFP<<hygr bub1::natr
Fig. S1A	SAK3672	h-	sgo2-3flag<<kanr
	SAK3745	h-	ade6 leu1 bub1::ura4+ sgo2-3flag<<kanr
	SAK3746	h-	ade6 leu1 hta1(S121A) hta2(S121A) sgo2-3flag<<kanr
	SAK3824	h-	sgo2-3flag<<kanr set2::natr
Fig. S1B	SAK3672	h-	sgo2-3flag<<kanr
	SAK3794	h+	sgo2(522TA)-3flag<<kanr
	SAK3797	h+	sgo2(63LP)-3flag<<kanr
Fig. S1C	SAK3672	h-	sgo2-3flag<<kanr
	SAK3972	h	sgo2-3flag<<kanr bub1::natr c::Padh1-ade6<<bsdr

Fig. S2B	SAK3905	h-	leu1 + pRep81-Sgo2(WT)
	SAK3906	h-	leu1 + pRep81-Sgo2(522TA)
	SAK3907	h-	leu1 +pRep81-Sgo2(522TA 63LP)
	SAK3908	h-	leu1 +pRep81-Sgo2(522TA 590KA)
	SAK3909	h-	leu1 +pRep81-Sgo2(63LP)
	SAK3910	h-	leu1 +pRep81-Sgo2(590KA)
	SAK3911	h-	leu1 +pRep81
Fig. S3	SAK856	h-	cnp3-tdtomato<<kanr sgo2-GFP<<hygr
	SAK3912	h-	cnp3-tdtomato<<kanr sgo2(522TA)-GFP<<hygr
	SAK3914	h-	cnp3-tdtomato<<kanr sgo2(63LP)-GFP<<hygr

Table S2. Primer list for ChIP

	forward	reverse
subtel	ACTCGCAACTGCTTTTTATG	TGTACGTTTCAAAGCAATGT
ostel-1	AAATCCGAAGTATGAGACAA	AAACATCCTTGTTTTTAACG
ostel-2	ATATTCATCGTCCAAGTAAA	CAAAGTTTTGATACCCTGAT
euch-1	AACGATTACATCTACGTCT	ATCAAAAGGACTATCTTAGA
dg	TTTTCAGCGAGACATGTACC	TCATAAAGCAACACTGGGTG
dh	GTAAGTATGAGCAACTGGCG	GGAACAAATCAGGAAACCGAG

Table S3 Primer list for RT-qPCR

	forward	reverse
gal1	GCGCCTTGTTTTGCTCTCC	ACGGGCGGTATGGATCAAT
gal10	AAGCAGTTGGGGAATCAATG	ACTGTCTTGACCCGGTGTTC
gal7	GATTTGGCTTCCGCATTAAA	AGCTTGGTGAAGACCCATTG
pan5	GTGGCCGGAAGAACAAAATA	TGGGATTCTTTGGGACTTTG
